# Relationship between blood and bronchial submucosal eosinophilia and reticular basement membrane thickening in chronic obstructive pulmonary disease

**DOI:** 10.1111/resp.12475

**Published:** 2015-01-27

**Authors:** Osama Eltboli, Vijay Mistry, Bethan Barker, Chris E. Brightling

**Affiliations:** ^1^ Institute for Lung Health, Department of Infection, Immunity & InflammationUniversity of LeicesterLeicesterUK; ^2^ Department of Medicine, Faculty of MedicineBenghazi UniversityBenghaziLibya

**Keywords:** blood eosinophil, chronic obstructive pulmonary disease, remodelling, reticular basement membrane, submucosal eosinophil

## Abstract

A sputum eosinophilia is observed in 10–40% of COPD subjects. The blood eosinophil count is a biomarker of sputum eosinophilia, but whether it is associated with bronchial submucosal eosinophils is unclear. In 20 COPD subjects and 21 controls we assessed the number of bronchial submucosal eosinophils and reticular basement membrane thickening and found these were positively correlated with the blood eosinophil percentage. In COPD, blood eosinophils are a good biomarker of bronchial eosinophilia and remodelling.

**Abbreviations**
COPD: chronic obstructive pulmonary disease
IL5: Interleukin‐5
MBP: major basic protein
RBM: reticular basement membrane and lamina reticularis

There is an increasing need to find non‐invasive reliable biomarkers in asthma and chronic obstructive pulmonary disease (COPD) to identify different phenotypes and direct treatment. A sputum eosinophilia (>3%) is present in 10–40% of COPD subjects.^1^ This biomarker and the intensity of eosinophilic inflammation in the bronchial submucosa predict the response to corticosteroid therapy in COPD.^1^, ^2^, ^3^ Alternative simpler biomarkers of eosinophilic inflammation are required. The peripheral blood eosinophil count and percentage are associated with a sputum eosinophilia^1^, ^4^ and can be successfully used to direct corticosteroid therapy in COPD.^5^ However, to date, studies have not explored the relationship between peripheral blood eosinophil count and the degree of submucosal eosinophils and remodelling in COPD.

We hypothesized that the bronchial submucosal (lamina propria) eosinophil count and thickening of the reticular basement membrane and lamina reticularis (RBM) is related to the peripheral blood eosinophil percent in COPD. To test our hypothesis, we analysed large airway bronchial tissue samples from COPD subjects and controls to determine the association between bronchial and blood eosinophil counts.

We undertook a single‐centre observational study at Glenfield Hospital, Leicester, UK. Subjects with or without COPD undergoing surgical lung resection for cancer or suspected cancer were recruited. COPD subjects (*n* = 20) were all ex or current smokers with spirometric evidence of airflow obstruction according to the Global Obstructive Lung Disease criteria for COPD. Subjects with a history of asthma were excluded. Control subjects (*n* = 21) included those with and without a smoking history without airflow obstruction. All subjects gave written informed consent. The study was approved by the Leicestershire, Northamptonshire and Rutland local ethics committee.

Bronchial tissue was dissected from the lung resection material and embedded in glycol‐methacrylate and stored at −80°C. Two micrometre sections were stained with monoclonal antibodies directed to major basic protein (Monosan; Newmarket Scientific, UK), tryptase (Dako, Ely, UK) for eosinophils and mast cells respectively and corresponding isotype controls (Dako). The number of eosinophils and mast cells were enumerated per mm^2^ submucosa (lamina propria between the RBM and airway smooth muscle bundle), and the RBM was measured as the mean of 50 measurements at 20 μm intervals. All measurements were undertaken by an observer blinded to the clinical characteristics.

Statistical analysis was performed using Prism version 6 (GraphPad, San Diego, CA, USA). In addition to comparisons between COPD and controls, the subjects were stratified into eosionophilh^igh^ and eosinophil^low^ determined by the group median peripheral blood eosinophil percentage as well as using the peripheral blood percentage cut‐off of 2% and an absolute peripheral blood eosinophil count of 0.3 × 10^9^/L.^1^, ^4^ Group comparisons were made using *t*‐tests and analysis of variance or Mann–Whitney and Kruskal–Wallis for parametric and non‐parametric analyses as appropriate and correlations by Pearson and Spearman rank tests. A *P* value of <0.05 was considered statistically significant.

The clinical characteristics are shown in Supplementary Table S1. There were no differences in gender between those with COPD and controls. COPD subjects were older and had a greater pack year smoking history and poorer lung function. The use of corticosteroids was similar between the eosinophil^low^ (3/10) and eosinophil^high^ (4/10) COPD groups (Fisher's Exact Test *P* = 1.0). There were no significant differences in the blood or submucosal eosinophil counts between COPD and controls nor in submucosal mast cell counts, although the RBM thickness was increased in COPD (9.7 (0.6)μm versus 7.1 (0.4)μm; *P* < 0.001) (Supplementary Table S1 and Fig. 1). The submucosal eosinophil count and RBM thickness was increased in the COPD eosinophil^high^ group (Fig. 1). Similar findings were observed with a cut‐off of peripheral blood eosinophil percent > 2% and absolute count > 0.3 × 10^9^/L (data not shown). In the COPD subjects, there was a strong correlation between the peripheral blood eosinophil percent and the submucosal eosinophil count (r = 0.57, *P* = 0.009) and RBM thickness (r = 0.59, *P* = 0.006) (Fig. 1). There was no correlation between the peripheral blood or bronchial submucosal eosinophil count and forced expiratory volume in 1 s or forced expiratory volume in 1 s /forced vital capacity ratio (data not shown).

**Figure 1:**
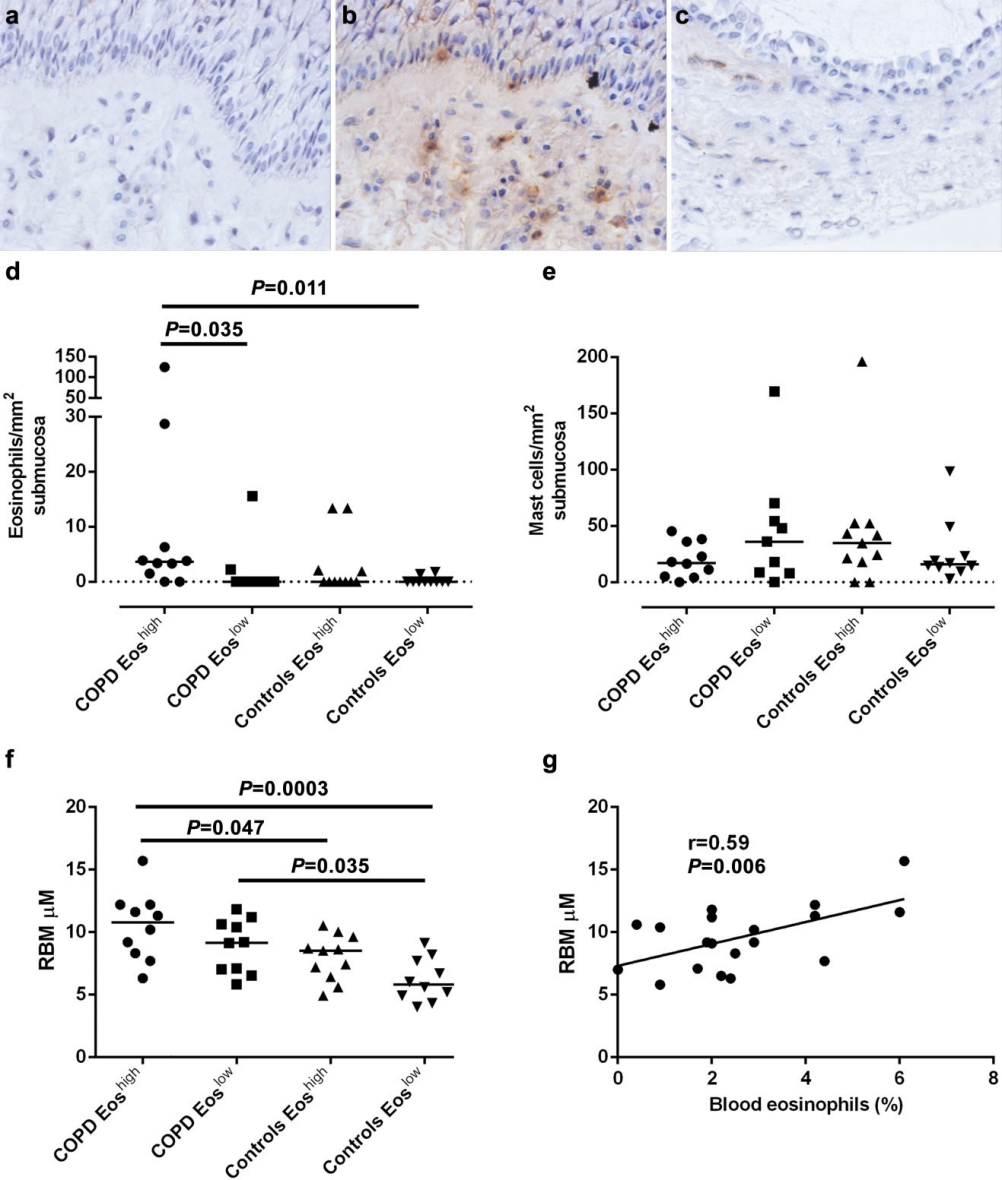
Representative bronchial tissue section stained with (a) isotype control, (b) major basic protein (MBP) illustrating eosinophilic inflammation and reticular basement membrane (RBM) thickening and (c) MBP showing and absence of eosinophilic inflammation and no RBM thickening (×400). Dot‐plots of (d) the submucosal eosinophil counts, (e) submucosal mast cells and (f) RBM thickness in the two chronic obstructive pulmonary disease (COPD) and two control groups, categorized by the median peripheral blood eosinophil count percentage. The horizontal bars represent the median comparisons by analysis of variance (parametric and non‐parametric as appropriate) and *P* values for post‐hoc tests as shown. (g) Scatter‐plot demonstrating the positive correlation between the peripheral blood eosinophil percent and RBM thickness in COPD subjects.

This study sheds light on the association between peripheral blood and submucosal eosinophils and airway remodelling in COPD. The correlation between peripheral blood and bronchial eosinophils and RBM thickening was strong and both the submucosal eosinophil count and RBM thickening were increased in the eosinophil^high^ group, suggesting that the peripheral blood eosinophil count does identify COPD subjects with a greater tissue eosinophilia.

Peripheral blood eosinophil counts are emerging as a valuable biomarker to phenotype airways disease and direct therapy. In the Dose Ranging Efficacy And safety with Mepolizumab study the peripheral blood eosinophil count correlated with response to the anti‐interleukin (IL)‐5 monoclonal antibody mepolizumab.^6^ The success of anti‐IL5 in subjects with severe asthma with evidence of eosinophilic inflammation^1^ raises the possibility that blood eosinophils might be an appropriate biomarker to direct eosinophil‐specific therapy in COPD. Indeed, a recent report does suggest that the peripheral blood eosinophil count identifies a subgroup of COPD subjects that have the greatest benefit in terms of lung function, health status and exacerbations in response to anti‐IL‐5‐receptor, Benralizumab.^7^

There were few potential drawbacks in our study. Baseline differences in age and smoking history between COPD and control groups might affect the differences observed in inflammation and remodelling. Additionally, we did not formally study whether subjects had atopy, and although we excluded subjects with asthma, we did not assess for asthma–COPD overlap syndrome. The study was retrospective, and contemporaneous sputum analysis was not performed. The number of subjects included was relatively small especially when they were divided into four groups based on blood eosinophil levels, although the correlations in the whole group were strong suggesting the findings were robust. However, further large‐scale prospective studies are warranted in order to validate and replicate our findings.

In conclusion, in COPD, peripheral blood eosinophils are associated with submucosal eosinophils and airway remodelling. Therefore, consistent with previous reports of the peripheral blood eosinophil count as a biomarker of a sputum eosinophilia, it is also likely to be related to airway tissue eosinophilia. These data further support the peripheral blood eosinophil count as an important biomarker of an airway eosinophilia and is likely to be important in targeting current and future anti‐eosinophil therapies for COPD.

## Acknowledgements

The authors sincerely thank all the research volunteers who participated in the study and wish to express their gratitude to the following people for their valuable assistance in the study: Rachid Berair, Aarti Parmar, Fiona Symon, Jane Middelton and Mitesh Pancholi.

This work was part funded by Medical Research Council and Wellcome Trust Senior Fellowship (CEB) and a sponsored studentship by the Libyan embassy in London on the behalf of Benghazi University in Libya. This paper presents independent research funded by the National Institute for Health Research (NIHR). The views expressed are those of the authors and not necessarily those of the NHS, the NIHR or the Department of Health.

## Supporting information


**Supplementary Table S1** Clinical characteristics of the COPD and control subjects stratified by the median peripheral blood eosinophil count percentage.
