# Supplementary material for: Relationship between blood and bronchial submucosal eosinophilia and reticular basement membrane thickening in chronic obstructive pulmonary disease
Source: Respirology. 2015 Jan 27;20(4):667–70. doi: 10.1111/resp.12475 (PMC4833195; doi:10.1111/resp.12475)
Supplement: Supplementary file 1 — Supplementary Table S1 Clinical characteristics of the COPD and control subjects stratified by the median peripheral blood eosinophil count percentage. [file RESP-20-667-s001.docx]

**Supplementary Information**

**The relationship between blood and bronchial submucosal eosinophilia and reticular basement membrane thickening in chronic obstructive pulmonary disease**

Osama Eltoboli MBBcH, MSc^1,2^, Vijay Mistry BSc^1^, Bethan Barker MBBS MRCP^1^, Chris Brightling BSc MBBS PhD FCCP FRCP^1^

^1^Institute for Lung Health, Department of Infection, Immunity & Inflammation, University of Leicester, Leicester,UK.

^2^Department of Medicine, Faculty of Medicine, Benghazi University, Benghazi, Libya.

**Table S1**: **Clinical characteristics of the COPD and control subjects stratified by the median peripheral blood eosinophil count percentage.**

|  | COPD  (n=20) | Control  (n=21) | P  value | COPD Eosinophil^high^  (n=10) | COPD  Eosinophil^low^  (n=10) | Control  Eosinophil^high^  (n=11) | Control  Eosinophil^low^  (n=10) | p  value | Pairwise  tests  p<0.05 |
| --- | --- | --- | --- | --- | --- | --- | --- | --- | --- |
| Gender (Male) | 17 | 18 | 1.0 | 9 | 8 | 10 | 8 | 0.82 |  |
| Age* | 67 (1.7) | 58 (1.8) | <0.001 | 69 (2.2) | 65 (2.5) | 55 (2.7) | 61 (2.0) | 0.003 | 6 |
| Smoking (pack years)* | 40 (20-50) | 4 (0-28) | <0.001 | 45 (20-50) | 40 (18-53) | 0 (0-34) | 8 0-25) | 0.009 | 3 |
| FEV_1_ | 1.94 (0.14) | 2.71 (0.12) | 0.001 | 2.06 (0.22) | 1.81 (0.18) | 2.84 (0.18) | 2.56 (0.16) | 0.001 | 2,4,5 |
| FEV_1_% predicted | 66.5 (3.8) | 83.8 (2.2) | 0.002 | 70.6 (4.7) | 62.4 (5.9) | 84.7 (3.3) | 82.9 (3.2) | 0.002 | 4,5 |
| FEV_1_/FVC% | 57.9 (2.9) | 76.5 (1.2) | <0.001 | \| 59.2 (3.9) \| \| --- \| \|  \| \|  \| | \| 56.6 (4.5) \| \| --- \| \|  \| \|  \| | \| 77.6 (1.7) \| \| --- \| \|  \| \|  \| | \| 75.3 (1.7) \| \| --- \| \|  \| \|  \| | <0.001 | 2,3,4,5 |
| Blood Eosinophils (%) | 2.7 (0.4) | 2.9 (0.4) | 0.65 | \| 4.0 (0.42) \| \| --- \| \|  \| \|  \| | \| 1.4 (0.25) \| \| --- \| \|  \| \|  \| | \| 4.3 (0.5) \| \| --- \| \|  \| \|  \| | \| 1.4 (0.12) \| \| --- \| \|  \| \|  \| | <0.001 | 1,3,4,6 |
| Blood Eosinophils x10^9^/L | 0.23 (0.03) | 0.22 (0.03) | 0.90 | \| 0.32 (0.05) \| \| --- \| \|  \| \|  \| | \| 0.14 (0.03) \| \| --- \| \|  \| \|  \| | \| 0.31 (0.04) \| \| --- \| \|  \| \|  \| | \| 0.13 (0.01) \| \| --- \| \|  \| \|  \| | <0.001 | 1,3,4,6 |
| Eosinophils/mm^2^ submucosa* | 0.75 (3.9) | 0 (1.6) | 0.09 | \|  \| \| --- \| \| 3.6 (10.8) \| \|  \| | \|  \| \| --- \| \| 0.0 (0.6) \| \|  \| | \|  \| \| --- \| \| 0.0 (2.1) \| \|  \| | \|  \| \| --- \| \| 0.0 (0.35) \| \|  \| | 0.009 | 1,3 |
| Mast cells/mm^2^ submucosa* | 18 (37) | 21 (32) | 0.70 | \|  \| \| --- \| \| 17 (34) \| \|  \| | \|  \| \| --- \| \| 36 (54) \| \|  \| | \|  \| \| --- \| \| 35 (34) \| \|  \| | \|  \| \| --- \| \| 16 (17) \| \|  \| | 0.45 | NS |
| RBM µm | 9.7 (0.6) | 7.1 (0.4) | <0.001 | \| 10.5 (0.9) \| \| --- \| \|  \| \|  \| | \| 8.9 (0.7) \| \| --- \| \|  \| \|  \| | \| 7.9 (0.5) \| \| --- \| \|  \| \|  \| | \| 6.2 (0.5) \| \| --- \| \|  \| \|  \| | <0.001 | 2,3,5 |

Data presented as mean (SEM), unless otherwise stated. *Median (IQR).

Abbreviations:FEV_1_: Forced expiratory volume in 1 second; FVC: Forced vital capacity; IQR: Inter-quartile range;NS: not significant; RBM: reticular basement membrane and lamina reticularis;SEM: standard error of the mean.

Post hoc pairwise comparisons p<0.05: 1- COPD eosinophil^high^versus COPD eosinophil^low^, 2- COPD eosinophil^high^versus control eosinophil^high^, 3- COPD eosinophil^high^versus control eosinophil^low^, 4- COPD eosinophil^low^versus control eosinophil^high^, 5- COPD eosinophil^low^versus control eosinophil^low^, and 6- control eosinophil^high^versus control eosinophil^low^.
